# Initial tweet valence, abuse volume, and observer Dark Tetrad characteristics influence perceptions of female celebrity abuse on Twitter

**DOI:** 10.1038/s41598-024-62273-y

**Published:** 2024-05-20

**Authors:** Christopher J. Hand, Joanne Ingram, Kayleigh Glover, Zara P. Brodie, Graham G. Scott

**Affiliations:** 1 School of Education, University of Glasgow, 11 Eldon Street, Glasgow, G3 6NH UK; 2 Applied Psychology Research Group, School of Education and Social Sciences, University of the West of Scotland, Glasgow, UK; 3 School of Health in Social Science, University of Edinburgh, Edinburgh, UK

**Keywords:** Human behaviour, Risk factors

## Abstract

Research into relationships between victim-generated content, abuse received, and observer characteristics when considering Twitter abuse has been limited to male victims. We evaluated participant perceptions of female celebrity victims and abuse received on Twitter. We used a 3 (Initial Tweet Valence; negative, neutral, positive) × 2 (Abuse Volume; low, high) repeated measures design and online survey method. Participants were shown tweets generated by six female celebrities, counterbalanced such that each participant saw each celebrity in one Valence-Volume condition. Stimuli were presented across six ‘lists’ such that celebrity ‘victims’ could be rotated across Valence-Volume pairings. Participants rated—per target stimulus—the level of blame attributable to the victim and the perceived severity of the incident. Furthermore, participants were asked to complete a Dark Tetrad scale—measuring their Machiavellianism, Narcissism, Psychopathy, and Sadism. Analyses determined that victim-blaming was influenced by victim Initial Tweet Valence (greater victim-blaming associated with more-negative content) and observer Machiavellianism. Perceived severity was influenced by victim Initial Tweet Valence, Volume of Abuse received, and observer Machiavellianism. Results were consistent with previous research involving male celebrity victims. Further research is needed to understand the contributions of participants’ hostile and benevolent sexism, as well as the role of victim attractiveness.

## Introduction

Victims are often blamed for acts perpetrated against them^1^. Two explanations for this are Just World Theory^2^ and Defensive Attribution^3^. Just World Theory suggests that people believe the world to be a just and fair place in which people ‘get what they deserve’. Thus, if harm is visited upon an individual then they must be deserving of it in some way. Defensive Attribution posits that individuals attempt to increase their sense of control by attributing harm that befalls others to the disposition of those individuals rather than to the environment they inhabit. Both defensive cognitions are designed to make individuals feel safe—victims of abusive acts brought harm upon themselves.

Victims of cyberabuse are often blamed for the abuse they receive^4^. Sympathy and support from family, friends, and authorities is often lacking^5,6^. Public perception is typically that online abuse is less harmful than offline abuse^7^. Often, the opposite is true^8^. Whereas offline abuse is sometimes fleeting and can perhaps be escaped from, abusive messages online are potentially permanent and can be shared with multiple audiences. It is hard for victims to escape negative content in online spaces without isolating themselves^9^.

Women receive proportionally more online abuse than men^10^. Women are vulnerable to image-based sexual abuse^11^. Women who express views about feminism and women’s rights are often targeted^12^. Compared to abuse directed to men, abuse against women on Twitter (now *X*; we use Twitter as this was current when our data was collected and is consistent with previous relevant literature) is likely to be sexist or misogynistic, and contain sexualized threats of violence, and many messages will come from individuals the victim has never met in real life^13,14^.

### Celebrity and the social network

Celebrities use social media to connect with fans and establish their brand^15^. Female celebrities are particularly visible on social media, with six of the ten most-followed accounts on Twitter belonging to women^16^. Female celebrities are powerful: Rhianna and Kylie Jenner caused Snapchat to lose up to $1 billion US after criticism of the company^17,18^. When celebrities receive abuse online it is often high-profile and can damage the public image upon which they rely, as well as negatively impacting them on a personal level^19^. Female celebrities receive more abuse than men^20^. Female sportpersons, for example, are sent explicit images by males online so frequently they say it is unrealistic to report all incidents to the police^21^. Several female celebrities left social media after receiving abuse^22^.

Research into which aspects of the online environment provide observers with cues that lead to victim blame and the downplaying of perceived severity has been driven by the Warranting Theory of online impression formation^23^. This proposes that there are two categories of online cues used to make judgments about individuals: identity claims (claims individuals make about themselves) and behavioural residue (unintentionally left evidence, including comments by third parties). Behavioural residue usually carries more weight in decisions as it is considered more objective and less self-serving^24^. Two factors which have been demonstrated to impact observers’ judgments of victim blame and perceived severity are the content produced by the victim (an identity claim) and volume of abuse received (a behavioural artifact^25,26^).

Scott et al.^26^ manipulated the valence of a tweet posted by a male celebrity (identity claim; negative, neutral, positive) as well as the volume of abuse received in response (behavioural residue; low, high). Male celebrity victims received most blame following a negative initial tweet, and least following a positive initial tweet; abusive incidents were perceived as more severe following a high volume of abuse. A similar pattern was also found for non-celebrity male victims^25^. Most blame was attributed to male Twitter users when initial tweets were negative, though in Hand et al.^25^ there was no difference between neutral and positive tweets. Hand et al.^25^ demonstrate that behavioural residue is an important cue for indicating perceived severity. Behavioural residue also contributes to perceptions of victim-blaming in lay-users. Identity claims are indicators of victim blame regardless of victim status. Hand and Scott^27^ examined the possibility that celebrities enjoy a protective ‘halo’, due to their perceived attractiveness. Celebrities were attributed less victim blame than lay-users; abuse targeted at celebrities was regarded as more severe. However, when celebrities tweeted negative initial content, they received more blame for abuse received. Victim-blaming was influenced by initial tweet valence, and perceived severity by both abuse volume and initial tweet valence.

The three studies detailed above^25–27^ considered only male victims. However, abuse against women is a significant problem and female celebrities are more likely to receive abuse than their male counterparts^20^. It is important to explore abuse of celebrity female victims to understand how they are viewed by those who would be either sympathise and offer support or potentially judge them negatively.

### The Dark Tetrad

The Dark Tetrad of personality (DT), reflecting subclinical variants of Machiavellianism, narcissism, psychopathy, and sadism^28^, has been implicated in both perpetration of online abuse and abuse perceptions. Psychopathy and sadism predict cyber-aggression perpetration, mediated predominantly by moral disengagement^29^. Machiavellianism, psychopathy, and sadism have been linked to enjoyment of online trolling behaviour and personal identification with trolls^30^.

Observer Machiavellianism, narcissism, and psychopathy predicted decreased perceived severity of abuse for both male celebrities and non-celebrity males on Twitter, while those high in narcissism and sadism demonstrate a higher propensity for victim-blaming^25,26^. Hand and Scott^27^ found that victim blame was predicted by observer Machiavellianism and psychopathy and that perceived severity was predicted by observer Machiavellianism. However, no research has yet examined observer DT traits in attitudes towards female victims of online abuse. DT traits and unsympathetic attitudes towards victims are argued to be underpinned by propensity towards sexist ideology^31^. Males and females who demonstrate high levels of DT traits exhibit elevated hostile and benevolent sexism^32^. Accordingly, the tendency to victim-blame and downplay perceived severity may be heightened in those with stronger DT characteristics.

### The current study

Participants processed six Twitter threads consisting of an initial tweet by a female celebrity followed by six replies. Initial tweets were either negative, neutral, or positive in valence. Replies included two (low volume) or four (high volume) abusive comments out of six. Participants indicated victim-blaming and perceived severity per target. Finally, participants completed a DT questionnaire.

Specifically, we predicted that *a negative initial tweet would be associated with greater levels of victim-blaming.* Female celebrities who tweeted negative content would be blamed more than celebrities who tweeted neutral or positive content. Related to this, we predicted that *Tweet valence would impact perceived severity of subsequent abuse; abuse following negative initial tweets would be perceived as least severe.* Abuse received by female celebrities who post negative content would be viewed by observers as less severe than ‘unprovoked’ abuse following neutral or positive content. Furthermore, we predicted that *A higher volume of abuse would be associated with greater perceived severity.* The more abuse female celebrities received, the more severe an incident would be perceived to be. Considering DT characteristics, we predicted that t*here would be positive associations between participants’ DT scores and attributed victim blame* and t*here would be negative associations between participants’ DT scores and perceived severity.* Participants scoring high on DT personality traits would generally demonstrate less empathy than others. Accordingly, they would minimize the harm and discomfort they perceive others to experience, while maximizing their defensive attributions.

## Results

### Data analysis

There were no missing values across profile ratings or DT measures. Two 3 (Initial Tweet Valence: negative, neutral, positive) × 2 (Abuse Volume: low, high) repeated measures analyses of variance (ANOVAs) were performed on victim blame and perceived severity ratings. Multiple linear regressions (stepwise method) explored predictors of victim-blaming and perceived severity—note. prior to this, Pearson’s correlations (one-tailed) identified relationships between co-variates and determined candidates for regression analyses.

### ANOVAs

Descriptive statistics for victim blame and perceived severity across conditions are presented in Table 1.

**Table 1:** Mean ratings (plus standard deviations) of victim blame (VB) and perceived severity (PS) with 95% confidence intervals across experimental conditions.

Valence	Volume	VB (SD)	VB 95% CI	PS (SD)	PS 95%CI
Negative	Low	3.13 (1.25)	[2.96–3.31]	2.95 (1.00)	[2.84–3.10]
	High	3.32 (1.28)	[3.14–3.50]	3.63 (0.94)	[3.50–3.76]
Neutral	Low	1.21 (0.50)	[1.14–1.28]	3.26 (1.05)	[3.11–3.41]
	High	1.24 (0.55)	[1.16–1.32]	4.00 (1.06)	[3.85–4.15]
Positive	Low	1.30 (0.63)	[1.21–1.39]	3.20 (1.05)	[3.05–3.35]
	High	1.26 (0.54)	[1.18–1.34]	3.97 (1.06)	[3.82–4.11]

Figures rounded to 2DP. Participant judgments were measured on 5-point scales with endpoints 1 (least blame/severity) and 5 (greatest blame/severity).

#### Victim blame

Mauchly’s test of sphericity was significant for the main effect of tweet valence (*W* = 0.346, *p* < 0.001; *ε* = 0.605). Greenhouse–Geisser corrections were applied. The main effect of initial tweet valence was significant and large [*F*(1.209,257.726) = 689.90, *p* < 0.001, *η*_*p*_^2^ = 0.779]. Planned follow-up comparisons demonstrated that victim blame associated with a negative initial tweet (3.23) was greater than victim blame following neutral tweets (1.22; *p* < 0.001) and positive tweets (1.28; *p* < 0.001); there was no difference between victim blame following neutral vs. positive initial tweets (*p* = 0.118).

The main effect of abuse volume on victim blame was non-significant [*F*(1,392) = 1.66, *p* = 0.200].

The assumption of sphericity was violated for the initial tweet valence × abuse volume interaction (*W* = 0.479, *p* < 0.001; *ε* = 0.657). Greenhouse–Geisser corrections were applied. This interaction was non-significant [*F*(1.315,257.726) = 2.34, *p* = 0.098].

#### Perceived severity

Mauchly’s test of sphericity was significant for the main effect of initial tweet valence (*W* = 0.929, *p* = 0.001; *ε* = 0.934). Huynh–Feldt corrections were applied. The main effect of initial tweet valence was significant [*F*(1.884,392) = 17.56, *p* < 0.001, *η*_*p*_^2^ = 0.082]. Planned follow-up comparisons demonstrated that perceived severity associated with a negative initial tweet (3.29) was lower than perceived severity following neutral tweets (3.63; *p* < 0.001) and positive tweets (3.58; *p* < 0.001); there was no difference between perceived severity attributed following neutral vs. positive initial tweets (*p* > 0.999).

The main effect of abuse volume on perceived severity was significant [*F*(1,392) = 247.85, *p* < 0.001, *η*_*p*_^2^ = 0.558]. Perceived severity was greater following a high volume of abuse (3.87) than a low volume of abuse (3.14).

The assumption of sphericity was upheld for the initial tweet valence × abuse volume interaction (*W* = 0.998, *p* = 0.860). This interaction was non-significant [*F* < 1].

### Regressions

Correlations between covariates which reached *p* < 0.10 were considered as candidates for multivariate models (as in Hand et al.^25^; Scott et al.^26^); typical significance limits (*p* ≤ 0.05) may fail to establish significance in dimensions otherwise known to be predictive^33^. Underlying correlations are summarised in Supplementary Materials II. Multicollinearity, independence of error terms, non-zero variances, normality, homoscedasticity, and linearity assumptions were upheld. Stepwise regressions were conducted for both victim-blaming and perceived severity.

#### Victim blame

A three-factor model was generated, with an *R* = 0.737 (adjusted *R*^2^ = 0.542) [Durbin-Watson = 1.936; *F*(3,1178) = 467.491, *p* < 0.001]. Co-efficients are detailed in Table 2.

**Table 2:** Victim blame regression coefficients.

Predictor	Unstandardized		Standardized			95% CI for B	
	*B*	Std. Error	*β*	*t*	*p*	Lower	Upper
Valence—neutral	− 2.006	0.061	− 0.745	− 32.781	< 0.001	− 2.126	− 1.886
Valence—positive	− 1.951	0.061	− 0.724	− 31.869	< 0.001	− 2.071	− 1.830
Machiavellianism	0.113	0.039	0.057	2.903	0.004	0.037	0.189

As victims’ tweets shift from negative to neutral, victim-blaming is significantly reduced; in turn, as initial tweet valence shifts from neutral to positive, again, victim-blaming is significantly reduced. Machiavellianism influences victim-blaming—participants who score more highly on this dimension attribute greater victim blame.

#### Perceived severity

A five-factor model was generated, with an *R* = 0.363 (adjusted *R*^2^ = 0.129) [Durbin–Watson = 2.027; *F*(4,1177) = 47.046, *p* < 0.001]. Co-efficients are detailed in Table 3.

**Table 3:** Perceived severity regression coefficients.

Predictor	Unstandardized		Standardized			95% CI for B	
	*B*	Std. Error	*β*	*t*	*p*	Lower	Upper
Volume	0.726	0.060	0.330	12.148	< 0.001	0.609	0.843
Valence—neutral	0.341	0.073	0.146	4.665	< 0.001	0.198	0.485
Valence—positive	0.292	0.073	0.125	3.988	< 0.001	0.148	0.435
Machiavellianism	− 0.107	0.046	− 0.063	− 2.317	0.021	− 0.198	− 0.016

A nuanced picture of perceived severity is presented. The volume of abuse received is particularly important in influencing ratings of perceived severity. As participants’ initial tweets move from negative to neutral, perceived severity increases, and as initial tweets move from neutral to positive, again there is an increase in perceived severity. As observer Machiavellianism increased, perceived severity typically decreased.

## Discussion

Our findings broadly supported all hypotheses. A complex interplay between victim-generated content (i.e., initial tweet valence), user-generated content (i.e., abuse volume), and observer characteristics (especiallyespecially Machiavellianism) shape perceptions of Twitter abuse involving female celebrities. This demonstrates both top-down (i.e., internalised) and up-bottom (i.e., stimulus-driven) risk factors in observer assessments of online abuse.

### Tweet valence and abuse volume

Victim-blaming was greatest when initial tweet valence was negative, supporting our hypothesis. According to Warranting Theory^23^ a tweet is a salient identity claim. There was no difference between victim-blaming across neutral vs. positive tweets, in line with Hand et al.^25^ who studied male lay-person victims. Observers attribute victims blame due to self-serving defensive attributions^3^. Our findings demonstrate that observers attribute more victim blame when they believe users have provoked abuse—tweeting abusive or negative content^26^. However, the current pattern of contrasts across levels of Initial Tweet Valence are somewhat different than those of Scott et al.^26^ (male celebrity victims) and Hand and Scott^27^ (male celebrities, male lay-persons), who found significant differences between all three levels of Initial Tweet Valence. These cross-study differences can be explained by a ‘crash’ in victim-blaming of female celebrities when tweets are anything-but-negative. With male victims (particularly male celebrities), previous victim-blaming data has shown a staggered, step-down across levels of initial tweet valence. This demonstrates a difference in how men and women are viewed online and merits further exploration.

Initial tweet valence impacted perceived severity. Perceived severity was lower in relation to negative than either neutral or positive initial tweets; there was no neutral vs. positive tweet perceived severity difference. These results support our hypothesis and dovetail with those involving male celebrities^26,27^. However, present results contradict Hand et al.^25^ who found no effect of Initial Tweet Valence on perceived severity for male lay-person victims. There appears to be something ‘special’ about celebrities—regardless of sex—and the victim-generated content and perceived severity association. Observers understand that celebrities’ use of social media may be more self-serving than lay-users’. Features of the online environment allow users to manage their personas more-carefully than in off-line situations (per the Hyperpersonal Model^34,35^); they can do this via identity claims. Consequently, not only may celebrities be blamed more for abuse received, but incident perceived severity might be lower^26,27^.

We predicted that a higher abuse volume would be associated with greater perceived severity, and this was supported. This aligns with previous studies involving male victims (celebrities, laypeople^25–27^). Behavioural residue is traditionally considered to be more accurate than identity claims, holding more weight in online impression formation (per Warranting Theory^23,24^). In this context, it contributes to observer impression formation and demonstrated that participants are not indifferent to abusive content and are capable of simultaneously recognising severity whilst also attributing victim blame. Results are consistent across victim sexes and status, suggesting that even though observers are aware of celebrities’ motives for using social media, they still recognise that they could be negatively impacted by abuse received.

### Observer Dark Tetrad

Positive associations between participants’ DT scores and victim-blaming were found, as hypothesised. When Initial Tweet Valence was neutral or positive, there were significant positive associations between all four DT dimensions and victim-blaming. When Initial Tweet Valence was negative, there was a negative relationship between observer Psychopathy and victim-blaming—as in Hand et al.^25^. It may be that observers with high psychopathy rather than identify with abusers instead ‘see themselves’ in the victim who has tweeted the negative content, and therefore attribute less blame because of defensive attributions^3^. It could also be that due to the lack of empathy that typifies individuals with higher psychopathy, they view the abusive tweet as acceptable and therefore deem the abusive reactions uncalled for.

Regression analysis on victim blame data revealed that user-generated content (i.e., Initial Tweet Valence) was the largest contributor in explaining victim blame variability; Machiavellianism also contributed to the model. As observers’ Machiavellianism increased, as did their likelihood of attributing victim blame. This is in line with Hand and Scott^27^. However, unlike previous research investigating perceptions of male victims, there was no place in the regression model of victim blame for narcissism (per Scott et al.^26^), sadism (per Hand et al.^25^), or psychopathy (per Hand and Scott^27^). These contrasting model structures suggest that there is a different interplay between victim-generated content and observer DT characteristics when victims are female as opposed to male.

Considering associations between observer DT scores and perceived severity, evidence was mixed, partially supporting our hypothesis. There was no evidence of a linear relationship between perceived severity and observer narcissism or sadism (n.b., this is consistent with certain results of Scott et al.^26^ [their Table 3], Hand et al.^25^ [their Table 4]. There was mixed evidence for a relationship between observer Machiavellianism and psychopathy on perceived severity; this depended on victim initial tweet valence. Regression analysis of perceived severity data revealed that abuse volume and initial tweet valence explained larger proportions of variability than observer characteristics; however, observer Machiavellianism significantly contributed to this model. This is in-part consistent with Hand et al.^25^ and Hand and Scott^27^. Unlike previous research investigating perceptions of male victims, models of perceived severity did not include narcissism (unlike Scott et al.^26^) or psychopathy (unlike Hand and Scott^27^; Hand et al.^25^; Scott et al.^26^). However, this does somewhat align with Lyons et al.^36^ who found that, of the three DT traits, those high in Machiavellianism alone were unable to correctly identify high-risk scenarios where females were likely to be sexually victimised. This suggests that those high in Machiavellianism fail to grasp the risk that online abuse may pose to female victims.

The current study used the 9-item Assessment of Sadistic Personality (ASP) tool^37^. These items were also used in similar research^25^. The 9 ASP items align with three theoretical origins—subjugation, pleasure-seeking, and non-empathy. However, other studies^38,39^ have utilised the 18-item Comprehensive Assessment of Sadistic Tendencies (CAST^40^). The CAST provides an overarching sadism score across three dimensions—verbal sadism, physical sadism, and vicarious sadism. Future research into the factors relevant in the current study may benefit from using the CAST to facilitate a more nuanced exploration of the independent and combined contributions of verbal, physical, and vicarious sadism.

### Implications for female users

Gender, and the assumptions that individuals hold about gender roles, may provide a particularly salient cue to individuals forming impressions in the relatively impoverished online environment (Social Identity Model^41,42^). When individuals behave outwith gender-sex roles expectancies they are often judged harshly and viewed as less competent by others^43^. This has been shown in online abuse towards female journalists^44,45^. By posting negative comments, female users could be perceived to have traversed the expected bounds of their gender-sex role and are therefore culpable for subsequently received abuse. More research is required to investigate this further.

Due to a general bias towards negative stimuli, viewers may attend to negative comments (abuse) more than positive comments^46^. In the context of online gender-sex role cues, online abuse may represent a social sanction against women^42^. This is relevant to the current study as even though exchanges containing a high volume of abuse were perceived to be more-severe, this does not necessarily mean that victims were believed to be undeserving of abuse.

Another salient cue is attractiveness, most obviously conveyed on social media via profile pictures (as well as conveying gender-sex). The current study neither manipulated the attractiveness of female celebrity victims, nor asked experimental participants about perceptions of victim attractiveness. However, data obtained from naïve participants during stimulus creation and norming suggests that our female celebrity ‘victims’ edged towards the positive (i.e., ‘attractive’) end of familiarity and feelings dimensions. Hand and Scott^27^ found that male celebrities were more socially-, physically-, and task-attractive than male laypersons, and that these attractiveness-es influenced victim-blaming and perceived severity. It may be that female celebrity victims were ‘attractive’ to participants, and this may have driven, for example, the ‘crash’ in victim-blaming when initial tweet valences were non-negative. Future research should explore the impact of victim attractiveness in relation to cyberabuse incidents involving female celebrities and female lay-persons, using carefully controlled stimuli.

### Limitations

One potential limitation of the current study was the sample composition—mainly younger adults and mostly women (our sub-sample of 32 men may not be representative of the broader population). Evidence suggests that males and females differ in DT characteristics. For example, psychopathy has been diagnosed more-frequently among males; in a forensic sample, 15–30% of males received a diagnosis of psychopathy, compared to between 9 and 23% of females^47^. Males generally score significantly higher in narcissism^48^. Machiavellianism is typically found to be higher among males^49^. The authors conducted analyses based on ‘gender’ with the current data set (we asked participants for their gender, rather than biological sex), and found no compelling evidence of independent effects nor interactions with other co-variates. Future research should carefully unpick contributions of participant sex and/or gender when considering perceptions of cyberabusive incidents and victims.

Replicating this work with a large, diverse, and representative sample is important to enhance the generalisability of the current findings. For example, ensuring appropriate representation of younger adults, middle aged, and older adults. It would be interesting to revisit the current issues with an adolescent population; however, this would present legal and ethical challenges, particularly given the provocative language necessary within the tweet stimuli. Ensuring representation of participants from across geographic and socio-economic diversities would strengthen work in this field, with a view to moving away from predominantly WEIRD representation.

We are confident that our tweet stimuli—which have been extensively normed as part of other projects^26^—are valid and realistic. However, it is a potential limitation of the work that participants were not looking at ‘real’ tweets inside Twitter itself. This may hinder the ecological validity of the study; however, it is a difficult balance to trade off the validity of interacting with dynamic stimuli in-app or on-site vs. the experimental control and rigour of off-line, carefully controlled stimuli.

## Conclusion

The current study is, to our knowledge, the first to examine the online abuse of female celebrities in this way. We found that female celebrities were only victim-blamed following negative initial tweets. Incidents were perceived to be severe unless initial tweets were negative. Observer Machiavellianism contributed to perceptions of both victim blame and perceived severity.

Although generally following the pattern of previous results investigating perceptions of abuse against male celebrities and male lay-users^25–27^, we identified subtle differences in observer impressions of abuse against female celebrities. Some of these can be explained by celebrities being viewed differently to lay-users and being viewed as more culpable for abuse they receive. Gender-sex differences may be explained by online perceptions of gender-sex roles and may be linked to victim attractiveness and observer benevolent sexism—more research is needed to explore this. Further research is required—working with victims, perpetrator, observers, technologists, legislators, etc.—to develop practical interventions to reduce offensive behaviour, encourage prosocial behaviour, and ensure that victims are supported appropriately.

## Method

### Participants

An a priori power analysis (G*Power 3.1.9.2) was conducted [F-test family, *α* = 0.05, desired power = 0.95^50^, smallest anticipated effect size = 0.15 (based on Scott et al.^26^]. Estimated target sample size was 150. Eventually, 197 participants completed all components of the study (164 women, 32 men, 1 non-binary participant; *M*_age_ = 30.01 years, *SD*_age_ = 7.96; range 18–68 years; median = 28 years; mode = 24 years). Participants were recruited through opportunity sampling entirely online between December 2020 and February 2021. Recruitment took place via advertisements on the researchers’ social media networks. Inclusion criteria included: native or proficient speakers of English (for non-native speakers, at least high school proficiency or equivalent) with no diagnosed visual impairments. Explicit exclusion criteria prohibited individuals under the age of 18 from taking part.

### Design and materials

We employed a quasi-experimental 3 (Initial Tweet Valence: negative, neutral, positive) × 2 (Abuse Volume: low, high) repeated measures design and an online survey method to explore participants perception of victim blame and perceived severity. Participants’ DT scores were recorded and associated with victim-blaming and perceived severity.

We created original victim profiles. To determine the names of the six female celebrity victims, we first established a ‘long list’ of 30 prominent female celebrities (actors, presenters, performing artists, sportspersons). The names of these celebrities were provided to an independent group of 27 participants who were asked to rate each for their familiarity (1 = not at all familiar, 5 = extremely familiar) and feelings associated with the named individuals (1 = very negative, 5 = very positive). We subsequently extracted six target celebrities (all active Twitter users in real-life) who were perceived as relatively neutral in terms of familiarity (each celebrity’s mean familiarity rating lay between 3.63 and 3.74; aggregated mean familiarity = 3.70, *SD* = 0.04) and feelings (each celebrity’s mean feeling rating lay between 3.44 and 3.85; aggregated mean feeling = 3.73, *SD* = 0.15). The six celebrity ‘victims’ extracted were Zoe Ball, Fern Britton, Tess Daly, Joanna Lumley, Jennifer Saunders, and Denise Van Outen.

Multiple sets of stimuli were created so that every celebrity victim was seen in each of the initial tweet valence × abuse volume conditions. These lists were counterbalanced and rotated such that each participant saw each victim only once yet saw a stimulus in each tweet valence × abuse volume condition.

Each participant saw six target stimuli, composed of an initial tweet by a female celebrity profile owner followed by six replies from Twitter users unknown to the participants. Stimuli were created using Microsoft Paint. Each stimulus consisted of (in order): celebrity ‘victim’ name and profile picture; their initial tweet; the number of comments, retweets, and favourites (numbers of which were controlled); then finally the six replies. Victims’ initial tweets were either negative, neutral, or positive, and within the six replies, either two (low volume) or four (high volume) were abusive. The written tweet content and replies (neutral and abusive) were identical to those of Hand et al.^25^ and Scott et al.^26^. An example stimulus is presented in Supplementary Materials I. Full details of the norming procedures for these tweets and replies are provided in Scott et al.^26^. Example tweets included: negative—“Isn’t it annoying that the really illiterate & rude people on Twitter are so fucking stupid that they forgot to kill themselves today.”; “You can't get anyone to do anything round here! Bunch of useless fucking c ∗  ∗  ∗ s!”; neutral—“Weathers getting chilly. I think summer is over”; “I'm in the mood to eat chocolate, lay on the sofa and do nothing …. That is all”; positive—“Be disciplined about doin’ the little things for your goals—daily. Consistency adds up to success. #ChaseYourGreatness”; “We are blessed to have another day to accept the challenge #GoCatchYourDream”. A full list of written content can be found within Scott et al.’s Appendix B^26^.

### Measures

To facilitate comparisons across studies, measures are similar to those of Hand et al.^25^. and Scott et al.^26^. Measures of victim blame and perceived severity were derived from Weber et al.’s^51^ direct and indirect victim blame. Four- and two-item measures using 5-point Likert-type scales were used to establish victim blame and perceived severity, respectively. An example item of the victim blame measure was: “Did the victim provoke the abuse?” (*1* = *strongly disagree*–*5* = *strongly agree*) and of the perceived severity measure was: “*How severe was the abuse?*” (*1* = *not severe at all–7* = *very severe*). Analyses revealed that both the victim blame and perceived severity measures were reliable [victim-blaming: Cronbach’s *α* = 0.936, *F*(3,1181) = 24.505, *p* < 0.001; perceived severity: Cronbach’s *α* = 0.822, *F*(1,1181) = 182.790, *p* < 0.001]. Victim-blaming and perceived severity measures were based on participants’ mean responses across items.

DT personality factors were measured by 36 items with a five-point Likert-type response scale (1 = *Strongly Disagree*–5 = *Strongly Agree*; 27 Dark Triad items of the SD3^52^; 9 sadism items from the ASP^37^). Example statements for each of the DT dimensions included: Machiavellianism—“You should wait for the right time to get back at people”; narcissism—“People see me as a natural leader”; psychopathy—“Payback needs to be quick and nasty”; sadism—“Being mean to others can be exciting.”. Cronbach’s alphas (*n*_*items*_ = 9) for Machiavellianism, narcissism, psychopathy, and Sadism were 0.689, 0.730, 0.730, and 0.635, respectively [all *F*s > 21.731, all *p*s < 0.001]. Each DT dimensional score was based on participants’ mean responses.

### Procedure

British Psychological Society^53^ principles were observed through the design and execution of this research. All methods were carried out per relevant guidelines and regulations. Approval was granted by the University of the West of Scotland School of Education and Social Science’s Ethics Committee. The survey was hosted by Qualtrics. Participants were given full instructions prior to providing informed consent. After reading task instructions, participants then provided their brief demographic data. For each stimulus, participants were asked to form an impression of the victim/initial tweeter and could view each target stimulus for as long as they wanted. Participants made victim blame and perceived severity judgements after processing each stimulus. After responding to all stimuli, participants then completed the DT survey before receiving full debriefing information. Participation lasted approximately 20 min.

### Declarations

British Psychological Society principles were observed through the design and execution of this research. All methods were carried out per relevant guidelines and regulations. Approval was granted by the University of the West of Scotland School of Education and Social Science’s Ethics Committee.

### Supplementary Information

Supplementary Information 1.

Supplementary Information 2.

## Data Availability

The data used in the analyses reported can be accessed via the Open Science Framework: https://osf.io/jd963/?view_only=1b3757bf0bec4e2db3c4604a19a1ed8a.
