# Supplementary material for: Initial tweet valence, abuse volume, and observer Dark Tetrad characteristics influence perceptions of female celebrity abuse on Twitter
Source: Sci Rep. 2024 May 20;14:11507. doi: 10.1038/s41598-024-62273-y (PMC11106073; doi:10.1038/s41598-024-62273-y)

**Supplementary Materials I**

**Example Stimuli**

**Negative Tweet – High Abuse Volume**


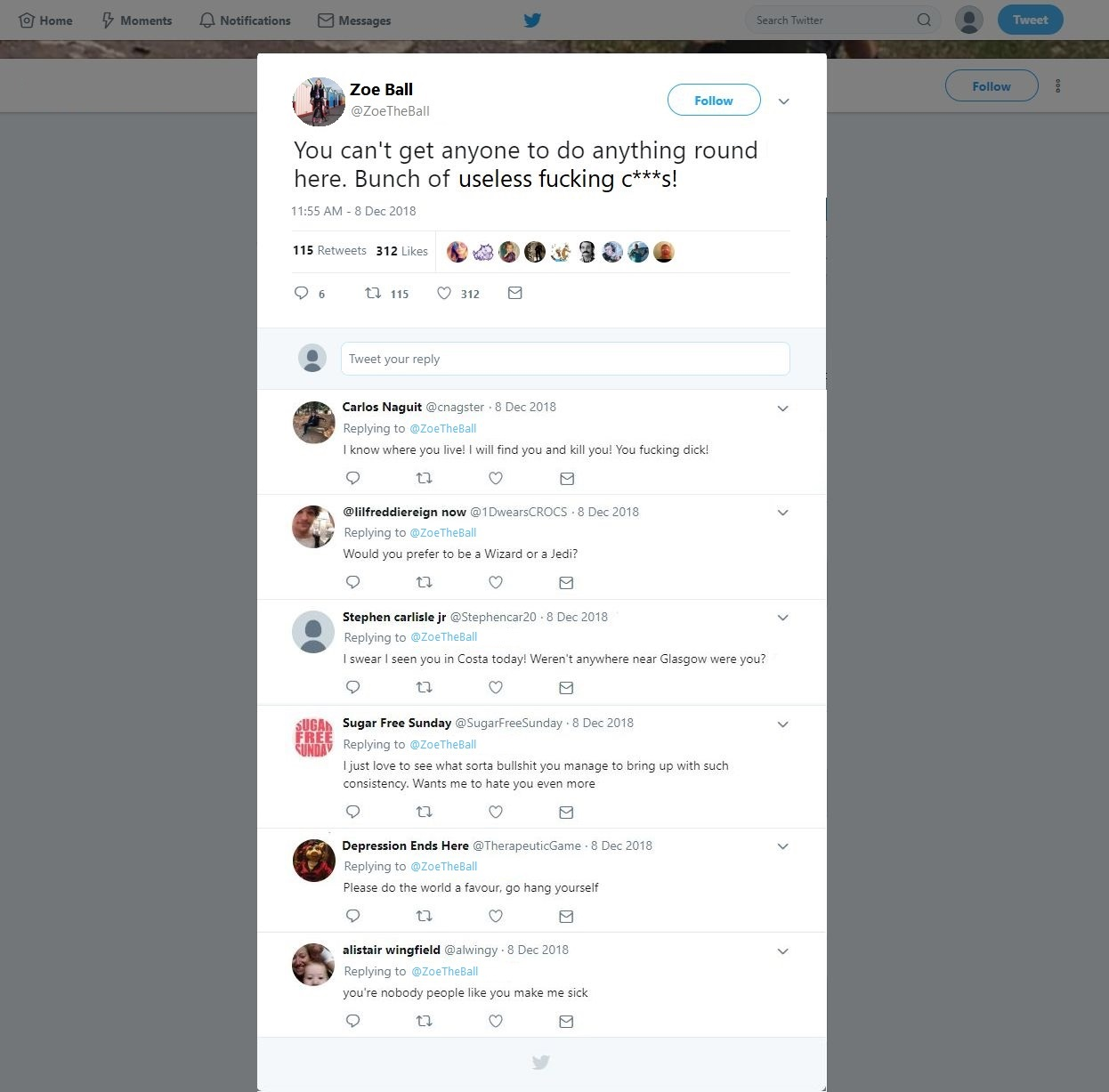


**Neutral Tweet – High Abuse Volume**


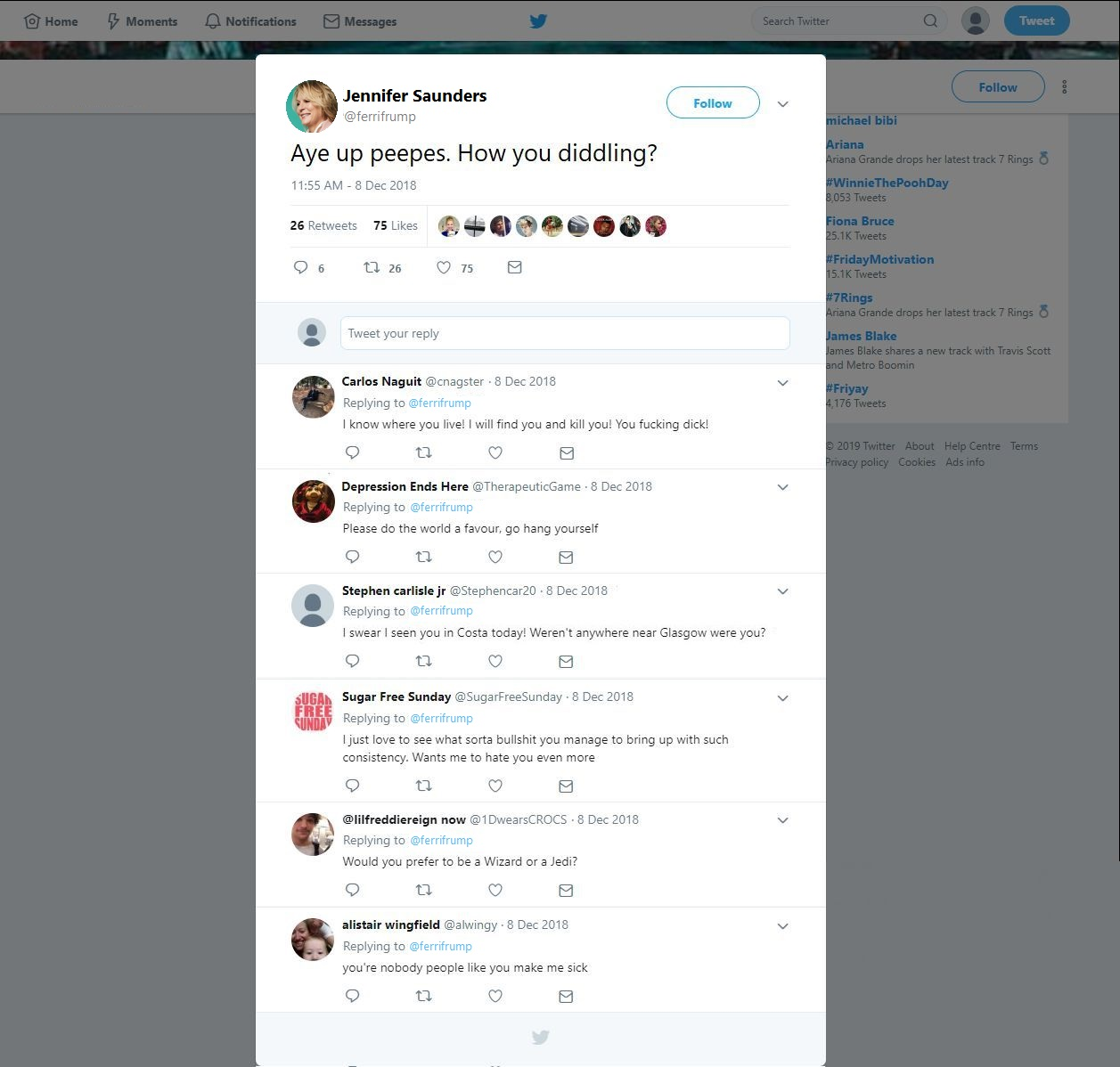


**Positive Tweet – High Abuse Volume**


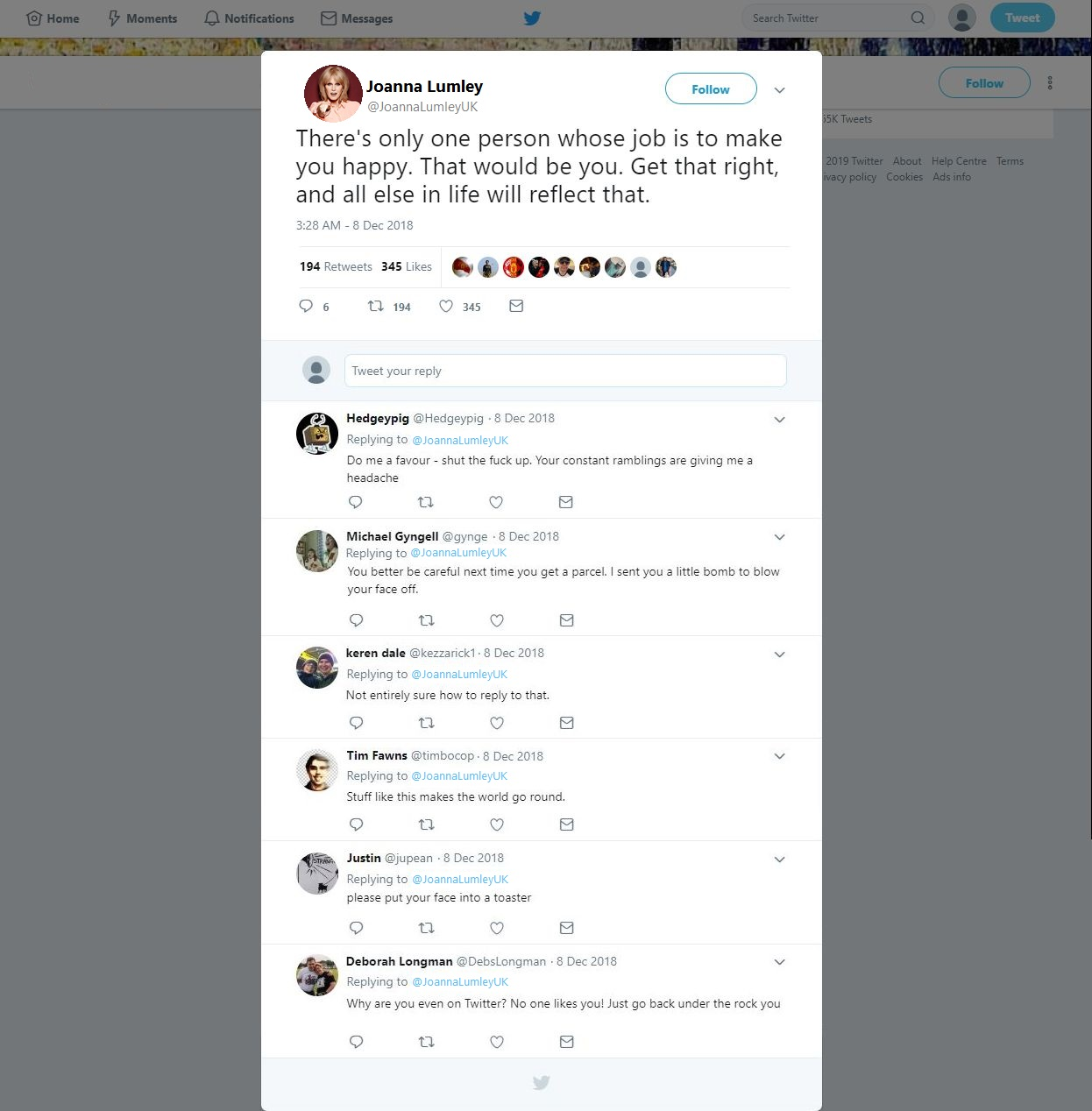

Supplement: Supplementary file 1 — Supplementary Information 1. [file 41598_2024_62273_MOESM1_ESM.docx]
