# Supplementary material for: Initial tweet valence, abuse volume, and observer Dark Tetrad characteristics influence perceptions of female celebrity abuse on Twitter
Source: Sci Rep. 2024 May 20;14:11507. doi: 10.1038/s41598-024-62273-y (PMC11106073; doi:10.1038/s41598-024-62273-y)
Supplement: Supplementary file 2 — Supplementary Information 2. [file 41598_2024_62273_MOESM2_ESM.docx]

**Supplementary Materials II**

**Correlations**

As the VB ANOVA revealed a significant main effect of Initial Tweet Valence only, correlations between participants’ DT scores, VB, and PS are partitioned by valence. Correlation coefficients and indicative significances are summarised in Table 4.

[Table 4 about here]

When initial tweets were negative, participants who score higher in psychopathy attribute less VB than those lower in psychopathy. When initial tweets were neutral, participants scoring higher in each of the DT dimensions attributed greater VB than those scoring lower on these dimensions. When initial tweets were positive, participants who scored higher in DT dimensions attributed more VB than those who were lower on these four dimensions.

The main effect of Initial Tweet Valence on PS was also significant, so again, correlations between co-variates are broken-down by valence and summarised in Table 5.

[Table 5 about here]

There were no associations between key covariates and PS when initial tweet valence was negative. Associated scatterplots supported an interpretation of ‘no relationship’, rather than a non-linear relationship which would not have been evident in the Pearson’s analyses. When initial tweets were neutral, participants who score higher in Machiavellianism and psychopathy were likely to report lower PS than participants who did not score highly on those two DT dimensions.

**Table 4.** Pearson’s correlations (one-tailed) among covariates by initial tweet valence – victim blame

|  | 2 | 3 | 4 | 5 | 6 | 7 |
| --- | --- | --- | --- | --- | --- | --- |
| 1. Machiavellianism | -.345** | .437** | .353** | -.041 | .200** | .164* |
| 2. Narcissism | -- | .454** | .463** | -.047 | .245** | .201** |
| 3. Psychopathy |  | -- | .672** | -.138* | .160* | .171** |
| 4. Sadism |  |  | -- | -.050 | .230** | .143* |
| 5. Negative Tweet VB |  |  |  | -- | .072 | .102 |
| 6. Neutral Tweet VB |  |  |  |  | -- | .710** |
| 7. Positive Tweet VB |  |  |  |  |  | -- |

*Note.* *n*=197. * *p*<.05, ** *p*<.01 (1-tailed)

**Table 5.** Pearson’s correlations (one-tailed) among covariates by initial tweet valence – perceived severity

|  | 2 | 3 | 4 | 5 | 6 | 7 |
| --- | --- | --- | --- | --- | --- | --- |
| 1. Machiavellianism | # | # | # | -.038 | -.146* | -.054 |
| 2. Narcissism | -- | # | # | -.022 | -.004 | -.005 |
| 3. Psychopathy |  | -- | # | -.031 | -.134* | -.070 |
| 4. Sadism |  |  | -- | -.019 | -.065 | -.078 |
| 5. Negative Tweet PS |  |  |  | -- | -.323** | -.461** |
| 6. Neutral Tweet PS |  |  |  |  | -- | -.618** |
| 7. Positive Tweet PS |  |  |  |  |  | -- |

*Note.* *n*=197. * *p*<.05, ** *p*<.01 (1-tailed). #=identical to data reported in Table 3.
